# Supplementary material for: Predictors of positive tuberculin skin test in neonates exposed to pulmonary tuberculosis
Source: PLoS One. 2024 May 9;19(5):e0303050. doi: 10.1371/journal.pone.0303050 (PMC11081389; doi:10.1371/journal.pone.0303050)
Supplement: S1 Table — * 1 HCW with a previous TB history was excluded † 1 HCW refused treatment ‡ 1 HCW stopped treatment due to side effect Abbreviations: IQR = interquartile range; HCW = health care worker; LTBI = latent tuberculosis infection; RFP = rifampicin; INH = isoniazid. (PDF) [file pone.0303050.s001.pdf]

S1 Table. Treatment results of contacts exposed to an index case with subclinical pulmonary tuberculosis

Variable	Status			Total
	Neonate	HCW	Household	
LTBI	8 (5.3)	9 (20.9)*	1 (16.7)	18 (8.7)
Treatment initiation	8 (100.0)	8 (88.9)†	1 (100.0)	17 (94.4)
Treatment completion	8 (100.0)	7 (87.5)‡	1 (100.0)	16 (94.1)
Treatment regimen				
RFP + INH (3 months)	3 (37.5)	6 (75.0)	0 (0.0)	9 (52.9)
RFP (4 months)	0 (0.0)	2 (25.0)	1 (100.0)	3 (17.6)
INH (9 months)	5 (62.5)	0 (0.0)	0 (0.0)	5 (29.4)
Observation period				
Person-months	2,665	1,018	126	3,809
Individual, median (IQR)	16 (16–20)	21 (16–21)	16 (16–21)	17 (16–21)
Progression to active tuberculosis during the observation period	0	0	0	0

* 1 HCW with a previous TB history was excluded

† 1 HCW refused treatment

‡ 1 HCW stopped treatment due to side effect

Abbreviations: IQR, interquartile range; HCW, health care worker; LTBI, latent tuberculosis infection; RFP, rifampicin; INH, isoniazid
