# Supplementary material for: Predictors of positive tuberculin skin test in neonates exposed to pulmonary tuberculosis
Source: PLoS One. 2024 May 9;19(5):e0303050. doi: 10.1371/journal.pone.0303050 (PMC11081389; doi:10.1371/journal.pone.0303050)
Supplement: Attachment — Submitted filename: Responses to Reviewers_PLOS ONE.docx [file pone.0303050.s002.docx]

**Reviewer #1**

**[General Comment]** In this article Song et. al., have assessed the TST positivity predictors in neonates exposed to TB index cases (healthcare workers) and undergone isoniazid prophylaxis regimen. As claimed by the author, this could become the first published study to find the predictors of positive TSTs among neonates exposed to an infectious index. The identification and characterization of the index cases and follow up success rate of this study should be appreciated. However, the study needs further clarity and major revision to justify the conclusion drawn from it.

**Response**: We appreciate the time and effort you dedicated to providing feedback on our manuscript and are grateful for the insightful comments and valuable improvements to our paper. We have gone through your comments carefully and tried our best to address them. The modifications in the manuscript have been highlighted in red. Here is a point-by-point response to your comments and concerns. We hope these revisions adequately address your concerns and contribute to our research's overall clarity and rigor.

**[Comment 1]** All the study subjects were exposed to index cases as defined in this paper. However, looking at the features of index cases (as in Table 1) and with the preventive measure taken for Covid-19 (Page 4, line 83), the likelihood of exposure is minimal. This has also been raised by the authors in the discussion (page 19, line 262). The authors should elaborate on the nature and likelihood of exposure.

**Response**: We appreciate your attention to detail and your engagement with the content. As mentioned in the manuscript, the facility had implemented enhanced infection prevention and control measures. These measures included maintaining physical distance, wearing surgical masks or other masks that offer greater filtration efficiency, following respiratory hygiene and cough etiquette, disinfecting the environment, and using mechanical ventilation.

As described in the manuscript, the three index cases worked approximately 8 hours per day, 4-5 days per week. Index cases A and B were primarily involved in newborn care, including feeding, changing diapers, and bathing newborns, while index case C primarily involved charting rather than newborn care. For most of the day, the mother fed her newborn. A mother and her neonate were allowed to spend two hours together daily in a mother’s room for two hours daily at Facility A and B. During this time, natural ventilation was implemented in the neonatal care room of Facility A. On average, the index patient was responsible for the care of about four newborns, and given the worker's usual work pattern, we estimate that they had about 30-40 minutes of close contact per day. Due to the nature of the neonatal care facility, physical contact between newborns and index patients could not be avoided; however, index patients without respiratory symptoms adhered to hand sanitization and mask-wearing when caring for newborns, thus minimizing the transmission of respiratory diseases. To address this concern, we will revise the method section to provide a more detailed explanation of the nature and likelihood of exposure as follows:

(Before) An index case at each facility is a nursing assistant who worked 8-hour shifts, 4 to 5 days a week. Their duties included caring for newborns in a neonatal care unit, which involved feeding, soothing, and changing diapers for approximately 5 to 6 hours per day. The index cases had casual contact with other nursing staff in the dressing room or during duty handover that lasted for approximately 30 minutes. (line 85-90 in the original manuscript)

(After) An index case at each facility is a nursing assistant who worked 8-hour shifts, 4 to 5 days a week. Their duties included caring for newborns in a neonatal care unit, which involved feeding, soothing, and changing diapers for approximately 5 to 6 hours per day. Most of the mothers fed their newborns during the day. A mother and her newborn were allowed to spend two hours together each day in a mother’s room with natural ventilation in the neonatal care room of Facility A during this time. Index cases A and B were primarily involved in newborn care, while index case C was primarily responsible for medical charting. On average, each index patient cared for approximately 4 newborns. Given the worker's typical work pattern, we estimate they had about 30-40 minutes of daily close contact with each newborn. The index cases had casual contact with other nursing staff in the dressing room or during duty handover that lasted for approximately 30 minutes. (line 85-96 in the revised manuscript)

**[Comment 2]** In the study, the percentage of TST positive cases among exposed indexes are 5.3%. If the objective was to investigate the impact of potential exposure in neonates on TST, then a cohort of neonates with no-exposure to any index cases will be useful for comparison.

**Response**: I appreciate your valuable insights and suggestions. First of all, as described in the manuscript, we would like to reiterate that this study aimed to identify various factors that may affect TST results, not just the duration of exposure. As you mentioned, a comparative study with newborns not exposed to TB patients would have provided useful information. However, in South Korea, in neonatal care facilities, the guideline [1] states that the scope and method of investigation are determined by the judgment of an expert committee, and all newborns who share the same space with the index patient during the presumptive contagious period, regardless of the exposure duration, are subjected to TST tests and management. In this study, all newborns were, to some degree, exposed to the index case, so the entire neonatal population in the facility was examined during the index case's presumptive period of contagiousness, making comparison of TST results with unexposed newborns in the 3 neonatal care facilities virtually impossible.

In addition to the analysis in Table 3, we further analyzed the predictors of TST positivity in newborns using the duration of exposure to the index case as a continuous variable and found that it was not statistically significant in logistic regression analysis (OR = 0.92, *P* = 0.621, 95% CI = 0.7-1.3), and there was no correlation between duration of exposure and TST size in correlation analysis (r = 0.04, *P* = 0.578). We also found no significant difference in exposure duration between TST positive and negative groups in the Student’s t-test (t = 0.49, *P* = 0.623). As you rightly pointed out, a cohort study with a control group that has no exposure will give us more useful information by mitigating the effect of potential bases or risk factors for TST-positive. We will cherish your insightful comment, and take it into account for future contact investigations and research.

|  |  |
| --- | --- |
| Exposure duration according to TST result  (Student’s *t*-test & Box plot; *P =* 0.623) | Scatter plot correlation of exposure duration and TST size  (Scatter plot for correlation analysis; (r = 0.04, *P* = 0.578 ) |

Reference

1. Korea Disease Control and Prevention Agency. Guidelines on tuberculosis management. Chungju, Republic of Korea: KDCA, 2023. Available: <https://www.kdca.go.kr/filepath/boardSyview.es?bid=0019&list_no=721583&seq=1> (Korean). Accessed November 6 2023

**[Comment 3]** The primary outcome of this study is- age (>< 6 days) at the initial exposure a predictor of TST reactivity. The chi-square test shows BCG vaccination as a determinants of TST positivity (univariate Firth coefficient test also showed some trend (0.08)). Also the Haldane-Anscombe correction emphasized on BCG vaccination as a critical factor (Page 15, line 222). Since the number of TST positive subjects among non-vaccinated cases is zero, it may over-fit statistical models used.

**Response**: Thank you for your thoughtful review and concerns. The Haldane-Anscombe correction is a valuable tool for stabilizing proportion estimates, particularly when dealing with extreme values and small sample sizes like our study [1-2]. It is important to note that the Haldane-Anscombe correction is not a multivariate analysis that controls for other factors, and when the multivariable analysis was performed using the logistic regression method, BCG vaccination was not a significant predictor at the 5 mm cut-off (*P* = 0.097). The effect of BCG vaccine on TST outcomes was difficult to conclude from our study. This is because univariable analysis (Table 3) using the Firth method and Mann-Whitney U test comparing BCG vaccine status and TST induration size (Fig 2. B) yielded significant results, but multivariable analysis did not. While we recognize the borderline significance, we have refrained from making definitive conclusions and emphasized the need for further research in this area.

Your observation regarding the potential overfitting of statistical models due to a zero count of TST-positive subjects among non-vaccinated cases is indeed crucial. We acknowledge the unique nature of our dataset, where there are no TST-positive cases among non-vaccinated individuals. This scenario could pose challenges in statistical modeling, and we appreciate your diligence in bringing attention to this matter. To mitigate the risk of overfitting and to enhance the robustness of our analysis, we, in fact, have used the Firth method. Firth regression is thought to be particularly valuable when the standard maximum likelihood estimation methods may lead to biased or inflated parameter estimates, especially when dealing with small sample sizes, separation issues, or rare events like ours [3]. When we tried to use conventional logistic regression to find predictors of TST positivity, the results were heavily skewed and difficult to interpret. We believe that the Firth method can mitigate the problems of small sample sizes and bias in conventional logistic regression estimates. In addition, we have performed additional statistical tests for cross-validation, including the Mann-Whitney U test, to further explore the relationship between BCG vaccination and TST induration size. This test provides complementary insights and contributes to a more comprehensive understanding of the observed trends. The manuscript also explicitly discussed the limitations associated with small sample size bias (line 286-288). We acknowledge the potential impact on model generalizability and have highlighted the need for cautious interpretation of results in light of this limitation as follows:

(Before) First, there were only 8 TST-positive neonates, a too-small number that decreases statistical power, although we utilized Firth logistic regression to minimize small-sample bias by penalizing odds ratios. Therefore, further study with an adequate sample size needs to be followed up to revalidate our findings. (line 286-289 in the original manuscript)

(After) First, there were only 8 TST-positive neonates with a zero count of TST-positives among the unvaccinated which can lead to biased or inflated parameter estimates, although we utilized Firth logistic regression to minimize small-sample and rare event bias by penalizing odds ratios. Therefore, with the need for a cautious interpretation of our findings, further study with an adequate sample size needs to be followed up to revalidate our findings. (line 300-305 in the revised manuscript)

Reference:

1. Haldane JBS. The mean and variance of χ2 when used as a test of homogeneity, when expectations are small. Biometrika. 1940;31(3/4):346-355. doi:10.2307/2332614
2. Lawson R. Small sample confidence intervals for the odds ratio. Commun Stat Simul Comput. 2004;33(4):1095-1113. doi:10.1081/SAC-200040691
3. Puhr R, Heinze G, Nold M, Lusa L, Geroldinger A. Firth’s logistic regression with rare events: accurate effect estimates and predictions?. Statistics in Medicine 2017; 36(14):383 2302-2317. https://doi.org/10.1002/sim.7273 PMID: 28295456

**[Comment 4]** The authors should elaborate on the BCG vaccination regimen used for the study subjects.

**Response**: As shown in the footnote† to Table 2, BCG vaccination has been administered intradermally (Danish strain 1331; Statens Serum Institue) and percutaneously (Tokyo strain 172; Japan BCG Laboratory) in South Korea. In this study, 92 out of 152 neonates were immunized with BCG, of which 53 were immunized intradermally, and 39 were immunized percutaneously. As per your suggestion, we have provided additional details on the BCG regimen in line 173 as follows:

(Before) Ninety-two (60.5%) neonates received BCG vaccination; 60 (39.5%) did not.

(After) Ninety-two (60.5%) neonates received BCG vaccination: 53 (57.6%) intradermally (BCG Danish strain 1331; Statens Serum Institut) and 39 (42.4%) percutaneously (BCG Tokyo strain 172; Japan BCG Laboratory). 60 (39.5%) did not receive vaccination. (line 185-187 in the revised manuscript)

**[Comment 5]** When were they vaccinated after birth?

**Response**: According to the Korean Standards for Immunization Practices and the National Standardized Immunization Schedule, BCG vaccination is recommended to be administered within 4 weeks of birth. If the vaccination is delayed, it is recommended to be administered until 2 months of age [1]. Patients in this study were vaccinated with BCG within 11 to 34 days (median 23 days, IQR 19-27) of birth.

Reference

1. Korea Disease Control and Prevention Agency. Guidelines and procedures for preventive vaccination. Chungju, Republic of Korea: KDCA, 2023. Available: <https://www.kdca.go.kr/filepath/boardSyview.es?bid=0019&list_no=722841&seq=1> (Korean). Accessed November 6, 2023

**[Comment 6]** Was there any heterogeneity in the time duration between vaccination and TST test?

**Response**: Thank you for your valuable feedback. The mean time from BCG vaccination to TST for neonates was 69 (± 11.0) days, with a range of 56 to 97 (median 69, IQR 63-74) days. We checked to see if there was any heterogeneity in the time between BCG vaccination and TST testing between the TST-negative and TST-positive groups. A chi-squared test for homogeneity at the third quartile (74 days) in the table below showed no statistically significant difference (χ² = 0.095, *P* = 0.713). A homogeneity test at the median or mean (69 days) also showed that the two groups showed no statistically significant differences (χ² = 0.457, *P* = 0.714).

|  | TST | | χ² | *p* |
| --- | --- | --- | --- | --- |
|  | Negative | Positive |  |  |
| Duration between BCG and TST | |  | 0.095 | 0.713 |
| <74 day | 57 | 27 |  |  |
| ≥74 day | 5 | 3 |  |  |

**[Comment 7]** Page 6, Table 1: Xpert MTB/RIF positive gives an impression of being Rif-resistant.
**Response**: Thank you for pointing out the confusion. All 3 index cases did not show RIF resistance in the XpertMTB/RIF test; we have reflected this in Table 1 of page 6 as follows:

(Before)

(After) (line 97 of Table 1 in the revised manuscript)

**[Comment 8]** Page 11, Table-2: Please clarify the missing 3% in prematurity [<37 weeks: 11 (5.4%), >37 weeks: 119 (91.6)] and 14.5% in delivery type.
**Response**: Thanks for pointing out the mistake. Out of a total of 152 newborns, 22 newborns in Facility B could not be assessed for prematurity and mode of delivery. We've made the following changes, which are also reflected in the Table 1 of the manuscript as follows:

(Before)

(After) (line 180 of Table 2 in the revised manuscript)

**[Comment 9]** Page 16, Table-3: Whether all the subjects completed the 3 months IPT regimen. What was the rationale behind setting the 68 days cut-off?

**Response**: The neonates (n=145) received IPT until they were 3 months old according to Korean Disease Control and Prevention guidelines [1], and the duration of IPT varied between subjects. The duration of IPT ranged from 8 to 78 days. The normality test (Kolmogorov-Smirnov test) did not satisfy the normal distribution (*P* = 0.045). Referring to the criteria suggested in another similar study [2], we set the third quartile (68 days) as the cut-off value and analyzed it in this study. For reference, the effect of IPT duration on TST positivity was not statistically significant when analyzed with the median (56 days) (χ² = 0.300, *P* = 0.723) or the mean (54 days) values (χ² = 0.054, *P* = 1.00). Multivariable logistic analysis to determine if IPT duration as a continuous variable had an effect on TST positivity was also not statistically significant (OR 1.098, *P* = 0.185, 95% CI 0.95-1.26).

Reference:

1. Korea Disease Control and Prevention Agency. Guidelines on tuberculosis management. Chungju, Republic of Korea: KDCA, 2023. Available: <https://www.kdca.go.kr/filepath/boardSyview.es?bid=0019&list_no=721583&seq=1> (Korean). Accessed November 6 2023
2. Yangthara B, Wutthigate P, Roongmaitree S, Siripattanapipong P, Lapphra K, Kitsommart R, et al. Nosocomial TB in two neonatal intensive care units at a tertiary care centre: infection risk and outcomes. The International Journal of Tuberculosis and Lung Disease. 2021; 25(7): 567–572.

**[Comment 10]** Page 16, Table-3: Percentage distribution <6 days 110 (99%) 1 (1%) and >6 days 34 (83%) 7 (17%) will give better impression on the data.

**Response**: Thank you for the valid point. We have made the following changes to line 242 of Table 3 as follows:

**Reviewer #2**

**[General Comment]** Author has attempted to see the predictors of positive TST in neonates exposed to Pulmonary Tuberculosis. Study is effectively planned and implemented. However, following points need clarifications:
**Response**: Thank you. We have incorporated your suggestions made by you. The modifications in the manuscript have been highlighted in red. Here is a point-by-point response to your comments and concerns. We hope these revisions adequately address your concerns and contribute to our research's overall clarity and rigor.

**[Comment 1]** Here index cases of health care workers have been detected by chest CT only. Is it routine practice to do TB screening with CT chest in author's setting? If no, author can explain what is routine practice and why CT were done in these cases.

**Response**: No. CT scan is not routine practice for TB screening in South Korea. A chest X-ray is the routine test for TB screening [1]. However, if the chest X-ray shows abnormal findings such as suspected tuberculosis or cancer, or if the results are unclear, a chest CT scan is performed at the discretion of the physician for a more accurate diagnosis. In our study, index cases A and C underwent chest X-rays as an annual examination and index B as a general health checkup. In the chest X-ray examination, index A and C had suspected tuberculosis (nodular lesion), and index B had unclear findings. All of them underwent additional CT scans according to the physicians’ judgment for more accurate findings.

Reference:

1. Korea Disease Control and Prevention Agency. Korean Guidelines for Tuberculosis, (fourth edition). Chungju, Republic of Korea: KDCA, 2020. Available: https://tbzero.kdca.go.kr/tbzero/contents.do (Korean). Accessed November 17, 2023

**[Comment 2]** In line no. 103, in index case A, how the nodule was detected? Is it on chest x-ray or CT scan?
**Response**: According to Article 11 of Korea's Tuberculosis Prevention Act [1], healthcare workers in neonatal care facilities are required to undergo a chest radiography test for tuberculosis every year. During an annual examination, Index case A was detected with a suspected pulmonary nodule in her right lung on CXR, leading to a chest CT scan at the discretion of the physician for a more accurate diagnosis. To clearly indicate the sequence of tests, we've made the following changes, which are also reflected in the manuscript (line 102-103).

Reference:

1. Ministry of Government Legislation. Article 11(Tuberculosis Examination, etc.) of Tuberculosis Prevention Act in South Korea. Sejong, Republic of Korea: MOGL, 2023. Available: <https://www.law.go.kr/LSW/eng/engLsSc.do?menuId=2&section=lawNm&query=Tuberculosis+prevention&x=0&y=0#liBgcolor1>. Accessed August 31 2023

(Before) During the annual TB examination on August 9, 2021, an asymptomatic 58-year-old nursing assistant was found to have a nodule in their right upper lung, but no cavities were detected.

(After) During the annual TB examination on August 9, 2021, an asymptomatic 58-year-old nursing assistant was found to have a nodule in the right lung, but no cavities on chest X-ray (CXR). At the physician’s discretion, a chest computed tomography (CT) scan was performed for an accurate diagnosis which confirmed the earlier findings, suggesting pulmonary tuberculosis. (line 108-111 in the revised manuscript)

**[Comment 3]** In line no. 105, for the same index case A, for which sample PCR or GeneXpert was done ? Is it sputum or biopsy sample?

**Response**: Index case A had difficulty in producing sputum, so she was unable to be tested with her own sputum. Instead, bronchoalveolar lavage (BAL) fluid was collected and examined for PCR and Xpert MTB/RIF. We've made the following changes, which are also reflected in the manuscript (line 104-105).

(Before) She had negative results for acid-fast bacilli (AFB) and cultures for *Mycobacterium tuberculosis*.

(After) Because of the scarcity of sputum, fiberoptic bronchoscopy was performed for the diagnosis. Her bronchoalveolar lavage (BAL) fluid showed negative results for acid-fast bacilli (AFB) and *Mycobacterium tuberculosis* cultures. (line 112-114 in the revised manuscript)

**[Comment 4]** Line no. 175, numbers are not matching. 152- 6 refusers - 2 refusers= 144. But author has written 142 completed the treatment.

**Response**: Thank you for pointing out the simple math mistake. Of 152 infants, 6 refused, and 2 dropped out of prophylactic treatment, leaving 144 infants to complete treatment by 3 months of age. We have reflected this in the manuscript (line 175 and Fig 1) as follows:

(Before) Of the 152 neonates, except 6 refusers and 2 drop-outs, 146 have completed 3 months of IPT.

(After) Of the 152 neonates, except 6 refusers and 2 drop-outs, 144 have completed 3 months of IPT. (line 189-190 in the revised manuscript)

(Before Fig 1-A)

(After Fig 1-A)

**[Comment 5]** Line No. 18, the author has commented that treatment was given to 18 contacts. But there is no mention of those 11 contacts detected positive for the first time by IGRA. What happened to those on follow-up? Were they also put on preventive treatment?

**Response**: The study team checked previous IGRA test history, treatment status, and TB notification through the Korean National TB Surveillance System (KNTSS). Of the 11 previous IGRA-positive individuals, 7 had completed latent TB treatment (6 with 3HR and 1 with 4R). Two individuals were registered in the system by verbally stating that they had completed latent TB or spinal TB treatment, while one individual was left untreated. The results of one individual are unknown. Of these 11 individuals, none were reported to have active TB since their last latent TB test or TB treatment. Reflecting on your comment, we have put the details of 11 individuals in the footnote and have added a symbol ^*^ in Fig 1 as follows:

(Before)

**Fig 1. Investigation flow chart and outcomes of (A) neonates and (B) health care workers exposed to an index case with pulmonary tuberculosis.**

^*^ Ten health care workers with negative results in the first IGRA were excluded from repeated IGRAs as the initial IGRA was provided 8 weeks after the end of the exposure

Abbreviations: CXR, chest X-ray; TST, tuberculin skin test; IGRA, interferon-γ release assay

(After) (line 144-151 in the revised manuscript)

**Fig 1. Investigation flow chart and outcomes of (A) neonates and (B) health care workers exposed to an index case with pulmonary tuberculosis.**

^*^ Seven had completed latent TB treatment. 2 health care workers were registered in the system by verbally stating that they had completed latent TB or spinal TB treatment, while 1 health care worker was left untreated. The results of 1 health care worker are unknown

^†^ Ten health care workers with negative results in the first IGRA were excluded from repeated IGRAs as the initial IGRA was provided 8 weeks after the end of the exposure

Abbreviations: CXR, chest X-ray; TST, tuberculin skin test; IGRA, interferon-γ release assay

(Before Fig 1-B)

(After Fig 1-B)

**[Comment 6]** Lines no. 220 and 236; in sensitivity analysis, author used 5 mm cut-off, but in methodology, author explained that 10mm cut off was chosen for those already taken BCG. Please explain why for analysis 5mm cut-off was considered. It may have given few false positive cases.

**Response**: Thank you for your thoughtful comments. The choice between 5 mm and 10 mm cut-off values in TST depends on the local epidemiology, patient population, and balance between sensitivity and specificity. A higher cut-off value reduces sensitivity and likelihood of overdiagnosis, but also reduces sensitivity and delays in diagnosis, leading to missing true positives.

While 10 mm is considered the cut-off for a positive TST in BCG-immunized individuals in South Korea, other countries like Germany, India, and European countries used 5 mm as a cut-off of TB positivity or to identify risk factors for TB infection, regardless of BCG status [1-4]. Given that the cut-off for TST positivity varies in children compared to adults [5,6], we conducted a sensitivity analysis, keeping in mind potential public health implications on TST cut-off adjustment, to compare whether there was a difference in the predictors of TST positivity when using 5 mm cut-off point in addition to 10 mm cut-off.

Reference:

1. Ferlinz, R. (1996). Guidelines for environmental contact tracing in tuberculosis. German Central Committee for control of tuberculosis. Gesundheitswesen 58(12):657–665.
2. Nayak, S., Acharjya, B. (2012). Mantoux test and its interpretation. Indian dermatology online journal, 3(1): 2-6.
3. Diel, R., Loddenkemper, R., Meywald-Walter, K., Niemann, S., Nienhaus, A. (2008). Predictive value of a whole blood IFN-γ assay for the development of active tuberculosis disease after recent infection with Mycobacterium tuberculosis. Am J Respir Crit Med 177(10): 1164-1170.
4. Diel, R., Loddenkemper, R., Niemann, S., Meywald-Walter, K., Nienhaus, A. (2011). Negative and positive predictive value of a whole-blood interferon-γ release assay for developing active tuberculosis: an update. Am J Respir Crit Med 183(1), 88-95.
5. Krishnamoorthy, Y., Ezhumalai, K., Murali, S., Rajaa, S., Jose, M., et al. (2021). Prevalence and risk factors associated with latent tuberculosis infection among household contacts of smear positive pulmonary tuberculosis patients in South India. Tropical Medicine & International Health, *26*(12): 1645-1651.
6. Campbell, J. R., Winters, N., Menzies, D. (2020). Absolute risk of tuberculosis among untreated populations with a positive tuberculin skin test or interferon-gamma release assay result: systematic review and meta-analysis. BMJ *368*.
