# Supplementary material for: Predictors of positive tuberculin skin test in neonates exposed to pulmonary tuberculosis
Source: PLoS One. 2024 May 9;19(5):e0303050. doi: 10.1371/journal.pone.0303050 (PMC11081389; doi:10.1371/journal.pone.0303050)
Supplement: Attachment — Submitted filename: Responses to Reviewers_v3.docx [file pone.0303050.s003.docx]

**[Journal Requirements]** Please review your reference list to ensure that it is complete and correct. If you have cited papers that have been retracted, please include the rationale for doing so in the manuscript text, or remove these references and replace them with relevant current references. Any changes to the reference list should be mentioned in the rebuttal letter that accompanies your revised manuscript. If you need to cite a retracted article, indicate the article's retracted status in the References list and also include a citation and full reference for the retraction notice

**Response:** Thank you for the advice on reference. We have double-checked the reference list and added DOIs in reference 12. Plus, we fixed the spacing in reference 21 and corrected a typo in reference 13 (“neonate” to “neonates”).

**Reviewer #3**

**[General Comment]** I appreciate the author (s) for coming up with this work on assessing the predictors of positive tuberculin skin tests in neonates exposed to pulmonary tuberculosis. It is an area with limited information and claimed by the author, it could be the first study in such a setting. We could build on this work for future studies. I am in support and acknowledged the work of other reviewers. However, the study needs to clarify minor corrections moving forward.

**Response**: We value your thoughtful suggestions and revisions you made to our manuscript. We also appreciate the time and effort you took to review our manuscript. After carefully reading your feedback, we have made every effort to respond to it. Based on the previously submitted manuscript, the changes in the manuscript have been highlighted in red. Here is a point-by-point response to your comments and concerns. With these adjustments, we expect to fully address your issues and improve the overall clarity and rigor of our study.

**[Comment 1]** Neonates are at risk of nosocomial tuberculosis (TB) infection from health care workers (HCWs) in neonatal care facilities, which can progress to severe diseases. The statement "which can progress to severe diseases." It sounds confusing as it may mean nosocomial tuberculosis (TB) infection from health care workers (HCWs) could progress to several other severe diseases than severe TB disease or different forms of TB.

**Response**: We appreciate your attention to detail and your engagement with the content. To clarify the meaning we originally intended, we have modified the sentences in the abstract and introduction as follows;

(Before) which can progress to severe diseases (line 22, Abstract in the original manuscript) → (After) which can progress to severe TB diseases (line 23 in the revised manuscript)

(Before) having the potential to develop invasive, fatal forms of diseases (line 52, Introduction in the original manuscript) → (After) having the potential to develop the most severe forms of TB such as TB meningitis or disseminated TB disease (lines 54–55 in the revised manuscript)

**[Comment 2]** The statement "BCG vaccination showed marginal statistical significance (Firth coefficient 2.5, 95% CI -0.4–5.4, P = 0.088) only in univariable analysis" in the abstract and result section could be use with caution. It is important to note that marginal significance differs from true statistical significance. Utilizing the term "marginally significant" is an indirect way of admitting that the resulting p-value was not statistically significant while attempting to give it the appearance of statistical significance. Researchers commit this fraudulent act because the more significant their results are (or, in this case, seem), the more likely the study is to be published; in turn, these publishments may reward researchers with grants or financial aid to continue their research or begin new studies (Lybrand et al., 2021).

**Response**: I appreciate your valuable insights and straightforward suggestions. Rather than overstating the statistical significance as you rightly pointed out, we would rephrase it in the following way;

(Before) BCG vaccination showed marginal statistical significance (Firth coefficient 2.5, 95% CI -0.4–5.4, P = 0.088) only in univariable analysis. (lines 36–38 in the original manuscript) → (After) BCG vaccination showed no statistical significance in both univariable and multivariable analysis (lines 39–40 in the revised version)

(Before) BCG vaccination is a marginally significant predictor (Firth coef. 2.5, 95% CI -0.4–5.4, *P* = 0.088). (lines 226­–227 in the original manuscript) → (After) BCG vaccination is not a statistically significant predictor (Firth coef. 2.5, 95% CI -0.4–5.4, *P* = 0.088). (lines 235–236 in the revised version)

(Before) BCG vaccination showed marginal statistical significance only in univariable analysis. (lines 249–250 in the original manuscript) → (After) BCG vaccination showed no statistical significance both in univariable and multivariable analysis. (lines 258–259 in the revised one)

We inserted the conjunction "however" in line 251 of the original manuscript to connect the sentences seamlessly. (line 260 in the revised manuscript)

(Before) Despite a marginally significant result in univariable analysis (line 263 in the original manuscript) → (After) Despite no significant result in univariable and multivariable analysis (line 272 in revised one)

(Before) our study reveals that age at the initial exposure is a statistically significant predictor of positive TST in neonates exposed to HCWs with pulmonary TB; BCG vaccination appears to be associated with positive TST. However, other risk factors, such as prematurity, sex, and exposure duration were not associated with positive TSTs. (lines 311–314 in the original manuscript) → (After) our study reveals that age at the initial exposure is a statistically significant predictor of positive TST in neonates exposed to HCWs with pulmonary TB. However, other risk factors, such as prematurity, sex, exposure duration, and BCG vaccination, were not associated with positive TSTs. (lines 321–324 in the revised one)

**[Comment 3]** While I appreciate the author's outstanding work describing the study area and settings, the author did not mention the study design in the abstract and methodology section. In addition, the author needs to be clear on sampling (sample size) and procedures.

**Response**: Thank you for your thoughtful review and suggestions. Investigations were conducted following TB exposure events in 3 different neonatal care facilities. To find factors that indicate a positive TST in newborns, relevant data, such as demographic, epidemiological, and clinical data, were gathered retrospectively.

This study can be labeled as ‘case-control’ in the sense that the primary study purpose is to compare two groups of people, TST+ neonates (case) and TST-neonates (control), and look back retrospectively to compare how frequently a risk factor (a predictor) is present in each group to determine the relationship between the predictors and TST positivity. However, we are somewhat hesitant to use the term case-control for our study design because 1) labeling our study a case-control study may be misleading as if we were identifying differences in risk factors based on the presence or absence of TB disease; 2) regrettably, differences in baseline characteristics between cases and controls are not fully accounted for; 3) although not a key question, progress to TB diseases among contacts, side effects of TB drugs, and treatment outcomes in our study are difficult to include in this category of case-control study; and 4) other TB contact investigation studies have rarely used the term case-control study. For these reasons, we prefer to describe our study design as a retrospective observational study. As you noted, we added a description of the study design to the Abstract and Methods section as follows;

(Before) Demographic, epidemiological, and clinical data of neonates exposed to TB, along with that of HCW and household contacts, were collected through contact investigations with the Korean National TB Surveillance System (KNTSS) database. TSTs using 2 tuberculin units of purified protein derivative RT23 were performed on exposed neonates at the end of preventive TB treatment. Firth logistic regression was performed to identify predictors of TST positivity. (lines 27–31 in Abstract) → (After) ) A retrospective observational study was conducted to compare the frequency of predictors between TST-positive and TST-negative neonates. Demographic, epidemiological, and clinical data of neonates exposed to TB, along with that of HCW and household contacts, were collected retrospectively through contact investigations with the Korean National TB Surveillance System (KNTSS) database. TSTs using 2 tuberculin units of purified protein derivative RT23 were performed on exposed neonates at the end of preventive TB treatment. Firth logistic regression was performed to identify predictors of TST positivity. (lines 28–34 in the revised manuscript)

We created the ‘**Study design’** subtitle right after the **Methods** section as follows;

**Study design**

Epidemiologic investigations were conducted following 3 TB exposure events in different neonatal care facilities. Details of the demographic, epidemiologic, and clinical data of exposed neonates, HCWs, and household contacts were collected retrospectively from investigation reports and the Korean National TB Surveillance System (KNTSS) database. We identified TST-positive and TST-negative neonates and compared the frequency of a risk factor in each group to determine the association between the predictors and TST positivity. (lines 71–77 in the revised manuscript)

(Before) The Korean National TB Surveillance System (KNTSS) database was used, (line 140 in the original) → (After) The KNTSS was used (line 149)

As you put it rightly, sampling procedures are important in TB contact investigation studies focused on identifying factors that predict a positive TST in newborns in neonatal care facilities. These procedures contribute to the study's representativeness, efficiency, statistical power, and generalizability, while also addressing ethical considerations associated with research involving newborns. However, the number of TST-positive cases, as well as the total number of exposed neonates reported or investigated in neonatal care facilities in our study, is too small, so sampling for representativeness, statistical power, and generalizability is difficult in practice. In fact, other papers on similar topics have not reached a sufficient number to consider the sampling procedure, and most of them were simple contact investigation studies in a single institution. As stated in the study limitations in the manuscript, we humbly acknowledge the sample size limitation. We cherish your points and intend to continue the study with careful attention to the sampling process in a follow-up study when we have a sufficient number of TST-positive newborns.
